# Bisphenol-A analogue (bisphenol-S) exposure alters female reproductive tract and apoptosis/oxidative gene expression in blastocyst-derived cells 

**DOI:** 10.22038/IJBMS.2020.40893.9664

**Published:** 2020-05

**Authors:** Alireza Nourian, Ali Soleimanzadeh, Ali Shalizar Jalali, Gholamreza Najafi

**Affiliations:** 1 Faculty of Veterinary Medicine, Urmia University, Urmia, Iran; 2 Department of Theriogenology, Faculty of Veterinary Medicine, Urmia University, Urmia, Iran; 3 Department of Basic Sciences, Faculty of Veterinary Medicine, Urmia University, Urmia, Iran

**Keywords:** Apoptosis, Bisphenol-S, Female reproductive tract, In vitro fertilization, Oxidative stress

## Abstract

**Objective(s):**

One of the major endocrine-disrupting chemicals, bisphenol-S (BPS) has replaced bisphenol-A due to public health anxiety. The present study evaluated low dosage BPS effect on female reproductive potential, hormonal disruption, and gene expression pathways of blastocyst-derived cells.

**Materials and Methods:**

NMRI female mice (5-6 weeks) in the estrous stage were chosen following vaginal smear examination for estrus cycle detection and BPS (0, 1, 5, 10, 50 and 100 µg/kg) was administrated subcutaneously for twenty-one consecutive days. After the last administration, blood, ovary tissue and oocytes were collected for further examination.

**Results:**

BPS induced oxidative stress in ovarian tissue and reduced hormonal status, LH and FSH, even at low concentration. Furthermore, apoptosis was induced in blastocyst derived cells in BPS administrated mice groups even at low BPS concertation, however, P53 and E2f1 expression were downregulated in doses more than 50 µg/kg, which might indicate apoptosis pathway exchange from P53 dependent to p53 independent pathways. IVF outcome was negatively associated with blastocyst apoptosis gene expression, estrogen receptor beta (ERβ) as well as oxidative status in ovaries. Finally, Stepwise regression indicated that E2f1, Nrf2, catalase (CAT), and malondialdehyde (MDA) could be chosen as predictor values for hatch percentage in IVF outcome.

**Conclusion:**

In summary, this study revealed BPS might have detrimental potential in the female reproductive system by oxidation induction and hormonal alteration as well as next generation blastocyst derived cells apoptosis induction. Further studies are recommended for public health assurance of BPS safety especially for female consumed products.

## Introduction

Modern lifestyle has increased human exposure to a variety of environmental toxins that enter the body via respiration and digestion (1). Endocrine disrupting chemicals (EDCs) are the major environmental pollutants that have been used routinely as the main component of many products such as plastics, food containers, cans, and pesticides (2).

Bisphenol-A (BPA) is one of the main EDCs that is widely used as an ingredient of hard polycarbonate plastics, epoxy resins and many other materials. BPA mimics an estrogenic effect by binding competitively to different types of estrogen receptors (3). Many studies investigated that not only BPA had detrimental potential for male and female reproductive system, but also, it decreased thyroid hormone level in serum, reduced hormone production (4, 5), reduced hypothalamic steroid hormone release (6), decreased male gonadotropin hormone level (7), abnormal embryonic development, and implantation induction (8, 9). Similarly, long-term administration of BPA disrupted the estrous cycle of non-pregnant mice (10) and ovarian reserve (11). Furthermore, another study revealed that low doses of BPA *in vitro* induced oxidative stress in testis (12-14). Due to public health concerns related to the toxic effects of BPA, its application is restricted especially in the US and replaced by “BPA-Free” products that contain substitutes such as bisphenol-F, bisphenol-B and bisphenol-S (BPS) (15).

As the most well-known BPA substitute, BPS is widely used in BPA-Free labeled products. After industrial replacement of BPA by BPS, the annual consumption of BPS has increased to more than 10,000 tons in Europe (16). Average absorption of BPS through foods was evaluated in a population living near the river and it was revealed that the estimated daily intake of BPS was 9 ng/kg of body weight (17). Recent investigations have detected the increased usage of BPS content materials in various countries which led to new concerns about health risks of BPS (18, 19). Recent studies show that BPS is more resistant in the ecosystem than mammalian organelles which increases its hazardous potential compared to BPA (20). 

BPS exposure hazardous influence on the reproductive system was detected recently. BPS influenced uterine weight increase, prenatal ovarian follicle development (21), maternal behavior, and lactation period (22, 23). Moreover, reduction in gonad weight, egg production, hatchability and embryo’s stages of development were reported in zebra fish that were exposed to low BPS and BPA dosages (24, 25). Alteration in hormonal activity and hypothalamic-pituitary-gonadal axis in males and females, even at the early stages of development were reported in exposure to different BPS concentrations (25-27). Furthermore, BPS obesity induction was reported in first and second generations by influencing the hypothalamic appetite pathway and deregulating lipid metabolism (20, 28, 29). Different metabolic pathways were detected in infants and pregnant mothers exposed to BPS that caused BPS metabolites abandonment in the infant body and could not be eliminated from them (30), In fact, BPS metabolized by conjugative rather than oxidative metabolism pathways (31). Moreover, an *in vitro *study revealed that oocyte meiosis maturation was interrupted by BPS even at doses lower than the dose human beings are exposed to the environment (32), and the effect of BPS on different embryonic stages was also reported in zebrafish (24). 

Like BPA, BPS induced oxidation-reduction at different doses in many tissues such as liver, kidneys, and testes. Moreover, influence of BPS on the male reproductive system was determined in different laboratory conditions (*in vivo* and *in vitro*), where BPS induced detrimental effects by increasing oxidative stress and decreasing testosterone concentration and daily sperm production (13, 33). Furthermore, prenatal exposure to BPS induced steroidogenic enzyme activity in female infants as well as oxidative stress and apoptosis in the male testis of next generation (34). 

BPA also induced reproductive system apoptosis by altering different cellular pathways (35, 36). To the best of our knowledge, no study was conducted to determine the detrimental effects of BPS on DNA and apoptosis in the female reproductive system. Based on our previous studies, BPS has a detrimental effect on *in vivo *fertilization capability as well as *in vitro* which was demonstrated by increasing type I arrest of IVF outcome (37, 38). 

This study was conducted to illustrate the effect of low concentration BPS on ovarian tissue oxidation-reduction induction, hormonal status disruption and different gene expression pathways of blastocyst-derived cells.

## Materials and Methods

***Chemicals***

Bisphenol-S (99%, 4, 4′-Sulfonyldiphenol) (CAS No. 80-09-1) and ethanol (ACS grade; CAS No. 64-17-5) were purchased from Sigma-Aldrich Company (St. Louis, MO, USA). The stuck solution was prepared by sufficient BPS powder dissolved in 100% ethanol, then, diluted in saline with less than 0.1-0.5% concentration of ethanol in the final solution (13).

***Animals***

All adult, cycling, female NMRI mice (5–6 weeks), which were in the estrous stage, were purchased and maintained in Urmia university animal house and the estrous cycle animals were chosen by vaginal smear (39). After seven days of adaptation, they were housed in standard glass cages with a 12- hr–12 hr light /dark cycle. Animals were fed phytoestrogens free diet with free access to glass water bottles.

***Experimental design***

**Figure 1:**
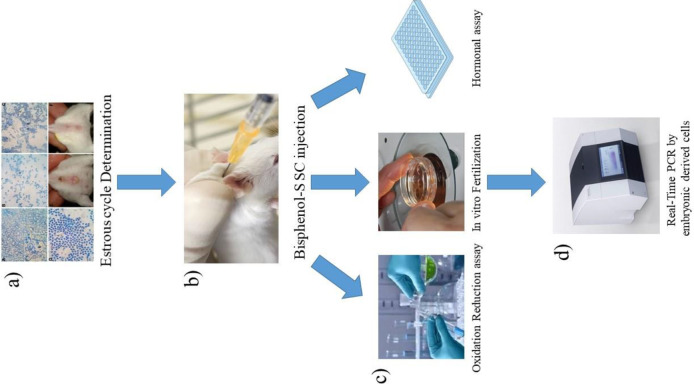
Schematic picture of the experiment. a) After estrus cycle detection all animals were divided randomly and b) BPS administrated Sc. c) IVF, Oxidative assay and ELISA were performed and d) Blastocyst cells harvested for real-time PCR

All steps were performed based on the National Institutes of Health Guide for Care and Use of Laboratory Animals and followed the principles of “Use of Animals in Toxicology”, with a slight modification, which were approved by Animal Ethics Committee (AEC) of University of Urmia (37). Briefly (Figure 1), different doses of BPS (0, 1, 5, 10, 50 and 100 µg/kg bodyweight/ day) were administered subcutaneously (SC) for eliminating the first-pass effect and increasing serum unconjugated to conjugated BPS ratio more than oral absorption (10) for 21 consecutive days. Three dosages lower than 50 µg/kg were selected according to the safe dose of BPA/day that was announced by the US Environmental Protection Agency (EPA) (40) .

One day after the last administration, blood samples were directly collected from hearts of the five mice of each group and centrifuged at 12000 x g for 10 min; then, mice were euthanized using Ketamine/Xylazine (45 mg/kg; 35 mg/kg; IP) (41) and ovarian tissue samples were harvested, which were immediately placed in phosphate buffer saline. The remaining mice were super-ovulated ( 5 mice per group) by pregnant mare’s serum gonadotropin (PMSG) and human chorionic gonadotropin (hCG) as described before (37). All oocytes were collected and inseminated by one normal mouse capacitated sperm. Four hours after insemination, all fertilized zygotes was transferred to the HTF medium and Blastocyst (10-20 in each group) was collected four days later. All samples were preserved in a refrigerator at -20 ^°^C for further evaluation. 

***Hormone level analysis***

Follicle-stimulating hormone (FSH) and luteinizing hormone (LH) level in serum were measured by Enzyme-Linked Immunosorbent Assay (ELISA) as described in the instructions provided by the kit manufacturer (Pishtaz Teb Diagnostics., Iran).

***Total RNA isolation and cDNA preparation***

Total RNA extraction was performed by the Choi method with slight modifications (42). Three pools of 20 unhatched (early blastocyst, blastocyst and expanded blastocyst stage) embryos were used, which were collected on the fourth day following *in vitro* fertilization (IVF). Subsequently, all embryos were lysed by RNX plus solution (Cinnagen, Iran) according to the manufacturer’s procedure. 1 ml of RNX solution was used to homogenize embryos and it was left intact at room temperature for 5 min. Later, 200 µl chloroform was added to the tube and centrifuged for 15 min at the rate of 12000 g at 4 ^°^C. RNA was obtained from the solution and an equal amount of isopropanol was added to it. The resulting compound was centrifuged again by the same procedure for 10 min. 75% ethanol was used to rinse the pellet and it was suspended again in 50 µl of Diethylpyrocarbonate (DEPC) treated water. Finally, total RNA concentration was examined by spectrophotometry (Thermo Scientific NanoDrop ND-2000 UV spectrophotometer,) at OD ≤1.9, 260/280 ratio. The RNA quantity and integrity were examined by the absorbance ratio A260/A280 nm and 1% agarose gel electrophoresis, respectively.

Equal RNA concentration (1 µg of DNase-treated total RNA) of each group was used for cDNA synthesis. cDNA Synthesis Kit Revert Aid was obtained from the Fermentas Corporation (Germany). One µg of RNA was reverse transcribed with 5X Reaction Buffer, 20 U/μl Ribolock RNase inhibitor, 10 mM dNTP, 200 U/μl MMLV reverse transcriptase, and oligo (dt)18 primer in a 20 μl reaction in order to synthesize cDNA. The resulting combination was incubated for 60 min at 42 ^°^C. Later, the enzyme was inactivated for 5 min at 70 ^°^C.

***Real-time PCR (RT-PCR) of different gene pathways of blastocyst-derived cells***

The integrated cDNA of all groups was further used in RT-PCR to reveal changes in the expression of the estrogen signaling pathway (*ERα* and *ERβ*) and apoptosis induction pathway (P53, Bax, Nrf2 and E2F1) genes. The details of gene specific sequence primers are shown in Table 1. All samples were run in triplicate and 18SrRBA was chosen as a house-keeping gene to normalize the input load of cDNA between samples. Hot Taq Eva Green (cat# BT 11101- SinaClon, Iran) chemistry was used for sample preparation. The reaction’s final volume equaled 25 µl , which included 1 µL of cDNA mixed with 1 µM primers (forward and reverse) for each gene. Thermal cyclings used for the reaction were as follows: initial denaturation at 95 ^°^C for 15 min, 40 cycles of denaturation at 95 ^°^C for 15 sec, primer annealing and extension at 62 ^°^C for 1 min. The fluorescence was seized at the end of the extension step. At the end of each round, a melt-curve analysis was performed to conclude the specificity of the amplification. The relative expression of each gene was examined using the delta CT model. For product homogeneity certainty, the melting curve analysis was assessed after the real-time PCR procedure.

***Examination of ovarian tissue oxidative activity***

Both harvested ovarian tissues were homogenized using a soft tissue homogenizer (Omni International, USA) in 9 volumes of ice-cooled 0.9% buffer saline solution. The homogenate was then centrifuged at 4000 x g for 10 min at 4 ^°^C and the supernatant was used for estimating antioxidant capacity. Ferric reduction antioxidant power assay was used for Total Antioxidant Capacity (TAC) assay according to Benzie and Strain’s study (43). Moreover, other anti-oxidant enzymes’ activities including superoxide dismutase (SOD), glutathione peroxidase (GPx), and CAT were determined as described by Nishikimi *et al*. Paglia and Valentine, and Sinha, respectively (44-46) and the activity of MDA was determined in our previous study (37). 

***Statistical analysis***

All analyses were performed using SPSS ver.24 (SPSS, Chicago, IL, USA) and data was stated as the mean±Standard Error (SE). Welch- one-way analysis of variance (ANOVA) test was used for comparison between groups and possible correlation was assessed by the Spearman test. *P*<0.05 was considered significant in all tests. Also multiple linear regression and stepwise regression were performed in order to determine the main indicator. Hatch percentage was selected as the dependent variable and all other variables including oxidative stress, hormone status and gene expression were chosen as predictors.

## Results

Note: There were not any malformation or organelle differences during the evaluation, hence, no animal was eliminated during the experiment. 

To investigate the dose-dependent influence of BPS on hormone levels, LH and FSH status were examined in which LH and FSH reduction was induced at the lowest administered doses. As represented in Table 2, 1 µg/kg of BPS caused a significant reduction in hormone status; interestingly, 50 µg/kg and 100 µg/kg had the same effect on LH and FSH hormone levels. Moreover, there were meaningful differences in LH and FSH status between two high dosage groups. 

As shown in Table 3, BPS induced oxidative stress in ovarian tissue even in the lowest dosage. All factors, except GPx, indicated significant oxidative stress in ovarian tissue induced by exposure to lowest BPS doses. Comparing the highest doses of BPS (50 µg/kg and 100 µg/kg), there were meaningful differences between all groups and only TAC status was constant.

**Figure 2:**
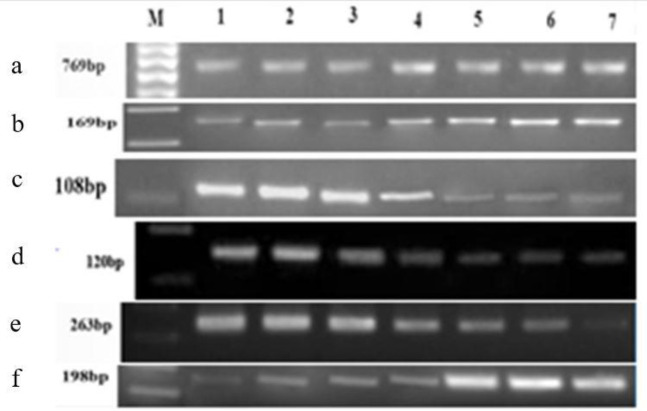
Gene's bands derived from cDNAs samples PCR using a) P53 (769 bp), b) Bax (169 bp), c) ERα (108 bp), d) ERβ (120 bp), e) Nrf2 (263 bp), and f) E2f1 (198 bp) gene primer; Lane M: 100 bp DNA ladder; Lane 1, control; Lane 2, B0; Lane 3, B1; Lane4, B5; Lane 5, B10; Lane 6, B50; Lane 7, B100 groups

Total RNA concentration detected by spectrophotometer ranged from 80 to 132 ng/μl for gene expression analysis. The Integrity and quality of all primers were examined by PCR which was verified by appearance of 769 bp, and 160 bp and absence of 1.3 kb DNA band on 2% agarose gel (Figure 2). In the present study, a different concentration of BPS was set for analysis of *P53*, *Bax*, estrogen receptor alpha (*ERα)*, *ERβ*, E2f1 and Erf2 genes by RT-PCR. It was found that expressions of *P53,* *Bax, ER*α, *ER*_β_ and E2f1 genes were significantly higher (*P*≤0.05) in doses of more than 5 µg/kg as compared to control groups. In comparison between 100 µg/kg and 50 µg/kg groups, there were significant differences in gene representation except for Nrf2. Interestingly, down-regulation was observed in P53 and F2f1 status in 100 µg/kg and 50 µg/kg groups which might indicate BPS-induced apoptosis pathways’ exchange from P53 dependent to p53 independent pathway in doses more than 50 µg/kg (Table 4). 

As seen in Table 5, all data had significant correlation with each other. A significant positive correlation was detected between all IVF parameters and all anti-oxidative enzymes except MDA. Significant negative correlation was observed between all examined genes and IVF parameters except ER*α*. A positive correlation was detected between LH, FSH, ER*α, *and all IVF parameters.

In multiple regression using ovarian oxidative stress, hormonal status and gene expression as predictors of IVF outcome, Nrf2, E2f1, MDA, and CAT were significantly associated with Hatch percentage (Table 6). The latter parameters’ predictive values were established by multiple logistic regression analysis and it was revealed that Nrf2, E2f1, MDA, and CAT could be used as indicators for IVF outcome prediction (Table 6).

## Discussion

Increasing use of BPS in new products raised public anxiety about its detrimental impact on human health. Recently, BPS was detected in blood and urinary samples of human beings with various ages (19, 47-49). Recent research indicates that after BPA, BPS is chosen as the second compound of bisphenol family which has the highest dermal absorption level from thermal paper (50). This study was conducted to illustrate the effects of exposure to the low concentrations of BPS on hormone disruption and oxidative factor status in mature females. It also aimed to define the exact mechanisms involved in next generation blastocyst-derived cells by evaluation of different gene expression pathways.

The levels of reproductive hormones (LH, FSH, testosterone, progesterone, and estrogen) have been reported to be changed by BPA exposure in different doses, length and method of administration (35, 51-55). Furthermore, the toxic effect of BPA on postnatal development was evaluated with an insignificant change of sex hormones (56). Based on our experiment, even at its lowest doses, BPS impaired LH and FSH production and there were significant differences between high-dose groups (50 µg/kg and 100 µg/kg), and it should be noted that 100 µg/kg of BPS induced higher reduction compared with other groups. Studies revealed that administering higher doses of BPS led to reduction of LH and FSH in female mice and this result is in agreement with the results obtained in this study (57). However, further studies are required for investigating the exact effect of BPS on hormonal production mechanisms. It should be noted that the effect of BPA on LH/FSH was previously established (35, 54, 55). Based on previous studies and the current study, it is suggested that exposure to low doses of BPS might have disrupted sex hormone secretion, production and performance potential in different animals and organelles. 

Interestingly, the effects of BPA and other members of the Bisphenol family on ROS production in different tissues were previously evaluated (58-60). The findings of our study also indicated that BPS increased oxidative stress status in ovarian tissue even in low doses by determining different antioxidant enzyme parameters including SOD, GSH-Px, CAT as enzymatic antioxidant and TAC as a non-enzymatic antioxidant detector. Besides, our previous study indicated that BPS increases lipid peroxidation, which is in line with the results of the present study (37). In addition, recent studies indicated that BPS increases oxidation potential in testis tissue and short-term BPS cultured spermatozoa (13, 33). Furthermore, oxidation induction of BPA and BPS might be related to their estrogenic potential especially because of their strong effect on estrogen receptor alpha (25, 61-63). The correlation between hormonal status and ovarian tissue oxidative stress delineated that BPS not only influences ovarian tissue as an oxidant, but also mimics the estrogenic effect that interrupts hormonal activity in the body.

The effect of BPA on pro-apoptotic and apoptosis gene expressions such as Bax and P53 as main indicators were previously established. Bax expression was up-regulated in response to BPA exposure in granulosa cells (64) and P53 expression was induced following 24hr BPA subjection in the ovarian cancer cell line (65) Furthermore, a correlation between BPA estrogen receptor stimulation and apoptosis induction was demonstrated by assessment of apoptotic genes in different *in vitro* cellular cultures (35, 36, 66). In this study, dose-dependent manner of p53 and Bax gene increase was induced in blastocyst collected from females exposed to different doses of BPS (Table 4). In agreement with our study, an *in vitro* study demonstrated that 40 mM of BPS in human adrenal cortico-carcinoma cells promotes P53 growth and apoptosis (67). Additionally, significant BPS dose-dependent apoptosis and DNA damage induction were detected in human testis tissue and human hepatoma cell line, respectively (61, 68), which confirms the results of the present study. P53 exact mechanism through which it influences embryos was previously explained. Briefly, the increase in P53 gene expression during early embryonic development induced Bax expression, as a result, it promotes apoptosis in the early stages of development. Consequently, embryonic arrest after apoptosis initiation could lead to embryonic lethality and abnormal embryo expansion (69). Additionally, embryonic hatch rate reduction and IVF outcome decrease were observed as mentioned before (37).

Estrogen Receptors (ERs) are involved in the early stages of fetal development and embryonic organ differentiation (70, 71). The increase in ERs has been demonstrated in brains exposed to BPA (72) , and the results indicate that BPA influences ERα gene expression and promotes apoptosis in breast cancer cells (66). A previous study demonstrated that in utero exposure of BPA during pregnancy influences ERα more than ERβ by mimicking estrogenic effect, therefore, it leads to adverse effects observed in early stages of development (73). Also, steroidogenic enzyme activity was increased by BPS prenatal exposure in next male generation testis and 50 µg/kg dosage group showed significant alteration compared to lower dosage in estradiol status and steroidal gene expression (34). Our study demonstrated that the effect of BPS on blastocyst-derived cells revealed P53 and ER expression and decreased ERα/ERβ ratio in response to the dose-dependent manner of BPS. Although, ER does not express after zygote fertilization until the blastocyst stage, it has a crucial role in blastocyst differentiation and implantation delay rate (74, 75). In agreement with our results, acute administration of BPS involved ER pathways especially primary macrophages and embryo-larvae in fish, however, up-regulation was detected in both ER genes expression (76, 77). However, dose and long-term response manner of BPS showed down-regulation in ERα expression in cell culture (78). Indeed, ERβ compared to ERα involved in long-term effects of ERs, and it elucidates the main cause of ERα reduction in the present study (77). It also suggested BPS selective specificity to ERα which significantly influences receptors in high dosage (79). Moreover, consistent with our study, long-term BPS administration down-regulated ERα expression in the ovary (21).

The E2f transcription factor family, is known as an essential part in the regulation of cell proliferation by mitosis checkpoint between G1/S phase (65), apoptosis induction in P53 dependent and independent pathway, and development in pre-implantation embryos (80, 81). E2f1 has been suggested to participate as the main p53-related apoptosis inducer (82-85), developmental stages of early embryos (80), and DNA replication malfunction in ovarian follicular and embryonic stem cells (86, 87). Based on the aforementioned studies, it is suggested that E2f1 has a crucial role in ovarian cancer and apoptosis. Our study showed that E2F1 gene expression was promoted in blastocyst cells in different BPS groups, which might indicate BPS teratogenicity and apoptosis- induced potential for infants even in low doses of administration. Interestingly, down-regulation was observed in the comparison of 50 and 100 groups and it probably indicates apoptosis pathway exchange from P53 dependent to P53 independent. Moreover, the same down-regulation was detected in P53 gene expression that is confirmed by pathway exchange hypothesis in 50 µg/kg. Our results are consistent with the study conducted by Cheraghi *et al*. who examined Zearalenone’s influence on E2F1 expression in which gene expression status was detected in moderate dosage (2 mg/kg) to be more than high dosage (4 mg/kg) in testis. They suggested reduction of E2f1 in the middle dose might be due to the apoptosis pathway exchange from p53 dependent to independent pathways (84). Possible side effects of low dosages of BPA (10 nM) were determined in ERα negative breast cell culture which apoptosis inducted by E2f1 dependent pathway. Also, a time-dependent manner and different cell culture examination showed the same results and suggested a possible ER independent side effect of BPA (88).

Interestingly, the correlation between ER and E2F1 in breast cancer cell line is a controversial issue. Some studies demonstrated a repressive effect between ER stimulation, especially ERα, and E2F1 gene expression (89), in contrast, stimulation of E2f1expression was observed by ER_α_ in normal MCF-7 breast cancer cell lines (90, 91) and cell lines that were tolerated to Tamoxifen (92). The correlation between ER and E2f1 was detected in our study, and positive correlation was detected between ER_β_ and E2F1, but negative correlation was detected between ER_α_ and E2F1. Due to positive correlation between E2F1 and P53 as well as previous investigations, it could be suggested that BPS administration induced apoptosis in blastocyst by N2f1/ p53 dependent pathway which is in agreement with Caspase 8 detection in cell culture exposed to different BPS concentrations (10^-14^ M and 10^-8^ M) (78). To the best of our knowledge, this is the first study to demonstrate the involvement of ER and E2f1 in different apoptosis induction pathways in blastocyst-derived cells that are harvested from BPS-exposed mother.

Nuclear factor erythroid 2-related factor 2 (Nrf2) is another transcription factor involved in embryonic development and mitosis entry. Nrf2 has multifunctional subgroup genes ,that participate in cytoplasmic defense against many environmental oxidants (antioxidant proteins) such as SOD, CAT, cell injuries by cyclin B-CDK1 complex and phase 2 detoxifying enzymes (93-96). Many previous studies presumed Nrf2 as an oxidant status indicator in pre-implantation embryos especially in blastocyst stage (93, 97, 98). BPA’s effect on lipid accumulation in the liver during pregnancy and in offspring was examined by Nrf2 as a pro-lipogenic factor. As a result, an increase in Nrf2 was reported both in pregnant mice exposed to BPA and their next generation offspring (99). The results of this study demonstrated that BPS induced significant oxidative stress that was indicated by Erf2 up-regulation in dosage higher than 10 µg/kg. Furthermore, no significant difference was observed between 50 and 100 µg/kg groups of BPS. A negative strong correlation was detected between Nrf2 and GPx, SOD, and Catalase which is consistent with the aforementioned hypothesis that signified Nrf2 as an indicator of oxidative stress.

Stepwise regression analysis revealed that E2f1, Nrf2, CAT, and MDA could be chosen as predictors of hatch percentage (Table 6) and current regression analysis failed to determine the meaningful relationship between other parameters with hatch capability. However, the Spearman coefficient determined meaningful correlation between all parameters and hatch percentages. 

**Table 1:** Details of primers (Primer sequence, amplicon size, and accession number) of different genes used in RT-PCR

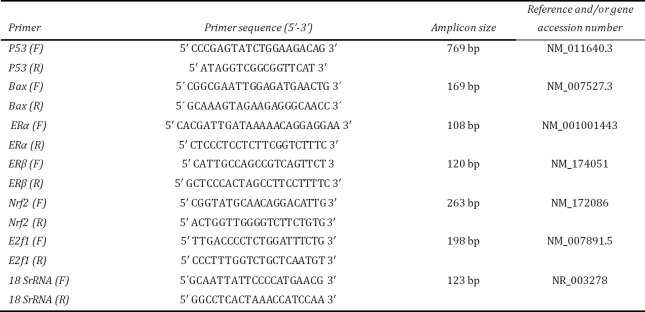

**Table 2:** Bisphenol-S (BPS) dose dependent effect on hormone status

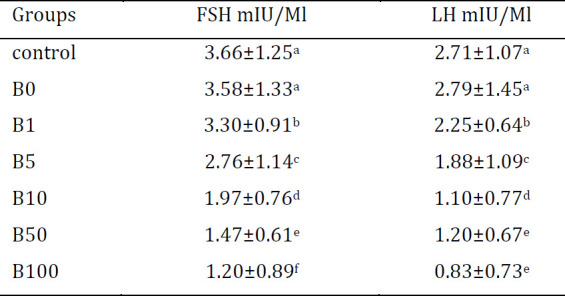

Different letters indicate significant differences between groups. Values represent means±SEM

BPS: Bisphenol-S; FSH: Follicle-stimulating hormone; LH: luteinizing hormone

**Table 3:** Different bisphenol-S (BPS) dose dependent oxidative induction in ovarian tissue

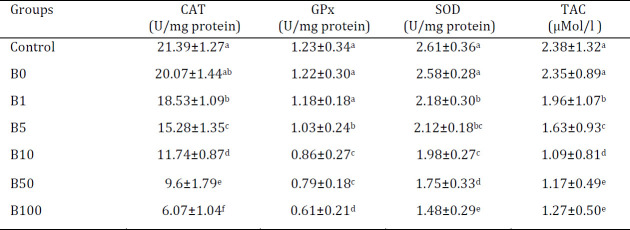

SOD: Superoxide dismutase; GPx: Glutathione peroxidase; CAT: Catalase; TAC: Total Anti-oxidant Capacity; BPS: Bisphenol-S; superoxide dismutase

Different letters indicate significant differences between groups. Values represent means±SEM

**Table 4:** Bisphenol-S (BPS) different dosage effect on level of mRNA expressions of genes (fold-change)

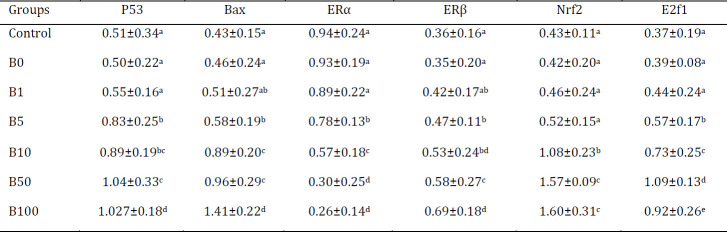

Different letters indicate significant differences between groups. Values represent means±SEM

**Table 5:** Spearman’s correlation coefficient between the results of the all experimental on blood, ovary tissue and oocytes of mice

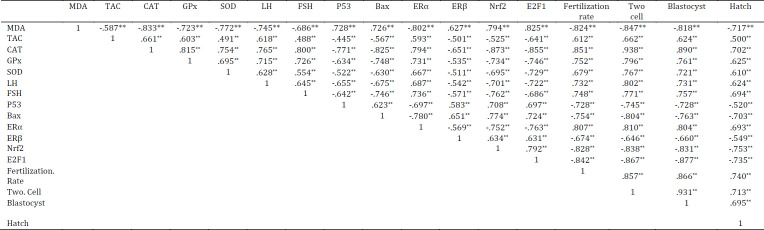

**Table 6:** Multiple linear and stepwise linear regression between ovarian oxidative stress, hormonal status and gene expression with Hatch percentage

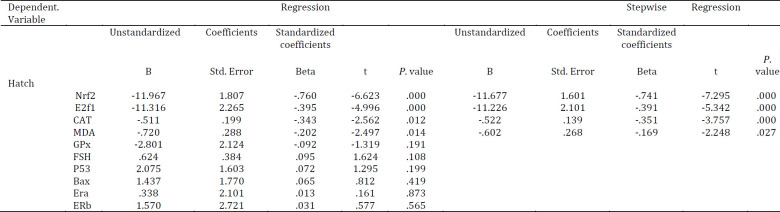

## Conclusion

The current study illustrates that low concentration of BPS had a detrimental effect on the female reproductive system by oxidative stress induction, gonadal hormone reduction, and next-generation apoptosis induction, which was determined by examination of Blastocyst-derived cells. Also, E2f1, Nrf2, CAT, and MDA have predictive value in the hatch blastocyst stage which can be used for pre-implantation embryo quality examination. Further studies are required to determine the exact mechanisms of BPS hormonal status as well as determining BPS capability for induced teratogenic effect on the next generations. 
